# Biomarkers for intestinal damage in pediatric celiac disease

**DOI:** 10.3389/fped.2026.2000459

**Published:** 2026-10-29

**Authors:** Kamola Sh. Usmonova, Svetlana I. Geller, Zulhumor E. Umarnazarova, Altinoy T. Kamilova

**Affiliations:** Department of Gastroenterology, Republican Specialized Scientific-Practical Medical Center of Pediatrics, Tashkent, Uzbekistan

**Keywords:** biomarkers, celiac disease, children, citrulline, gluten immunogenic peptides, gluten-free diet, intestinal fatty acid-binding protein, zonulin

## Abstract

Celiac disease (CD) is an immune-mediated enteropathy triggered by gluten in genetically predisposed individuals, affecting around 1% of the global population. Diagnosis relies on serological testing (IgA antibodies to transglutaminase 2 and endomysium), supported by HLA-DQ2/DQ8 typing and duodenal biopsy when indicated. This approach has limitations, including false negatives in IgA deficiency, poor sensitivity in potential or minimal-lesion CD, poor correlation with mucosal healing, and limited ability to predict disease progression. This mini review summarizes non-invasive and minimally invasive biomarkers for the diagnosis and follow-up of CD, with special attention to the pediatric setting. We discuss markers of enterocyte injury (intestinal fatty acid-binding protein, i-FABP; citrulline), immune activation (cytokines, high-mobility group box 1), gluten exposure and barrier function (fecal and urinary gluten immunogenic peptides, zonulin, calprotectin), and molecular signatures emerging from microbiome and multi-omics research. Of these, i-FABP (for diagnosis and follow-up) and gluten immunogenic peptides (for monitoring adherence to a gluten-free diet) appear the most promising. However, assay standardization, age-specific reference ranges, and large-scale prospective pediatric validation remain necessary before routine implementation, and data from regions such as Central Asia are scarce.

## 1 Introduction

Celiac disease (CD) is an immune-mediated enteropathy triggered by gluten in genetically susceptible individuals carrying HLA-DQ2 and/or HLA-DQ8. Once considered predominantly European, CD is now recognized as a global problem, with an estimated prevalence of 1%–1.5% and a rising incidence in children (1, 2). Epidemiological data from Central Asia remain limited. A systematic review and meta-analysis of the Asia-Pacific region reported a pooled seroprevalence of celiac disease of 1.2% in low-risk populations, with a higher prevalence observed among children; however, the authors highlighted the lack of population-based data from several Central Asian countries, including Uzbekistan, Kazakhstan, Kyrgyzstan, Tajikistan, Turkmenistan, and Mongolia (2, 3). The first prospective pediatric study from Uzbekistan identified celiac disease in 11 of 206 symptomatic children (5.34%), providing preliminary evidence of a substantial disease burden in this selected clinical population, although these findings cannot be extrapolated to the general pediatric population (4).

In children, CD presents with a broad clinical spectrum, ranging from classical gastrointestinal manifestations (chronic diarrhea, abdominal distension, malabsorption, failure to thrive) to atypical and extraintestinal features such as iron-deficiency anemia, short stature, delayed puberty, reduced bone mineral density, dental enamel defects, and neurological symptoms (5, 6). This heterogeneity frequently causes diagnostic delays, particularly in oligosymptomatic children (7).

Current diagnostic algorithms recommended by the European Society for Paediatric Gastroenterology, Hepatology and Nutrition (ESPGHAN) combine clinical assessment with serology [IgA antibodies against transglutaminase 2 (anti-TG2 IgA) and endomysial IgA antibodies (EMA-IgA)] and, only when indicated, duodenal biopsy; in selected patients with anti-TG2 IgA ≥10 times the upper limit of normal and positive EMA-IgA, biopsy may be omitted (8). HLA-DQ2/DQ8 typing offers high negative predictive value but limited specificity given its prevalence in the general population (8, 9). Diagnosis remains particularly challenging in IgA deficiency, minimal mucosal lesions, potential CD, and seronegative disease, where conventional serology may be insufficient (10).

Lifelong adherence to a gluten-free diet (GFD) is currently the only effective treatment, yet available serological markers correlate poorly with histological recovery and may miss low-level gluten exposure or ongoing epithelial damage (11–13). Advances in immunology, microbiome research, transcriptomics, and molecular diagnostics are creating new opportunities for a better diagnostic approach, with emerging biomarkers for epithelial injury, permeability, immune activation, gluten exposure, and host-microbiome interactions poised to complement conventional serology, particularly in pediatric patients (14, 15).

This mini review summarizes recent advances in emerging biomarkers for pediatric CD, focusing on their potential role in early diagnosis, disease monitoring, and precision medicine. Sources were identified through a narrative search of PubMed from January 2002 to June 2026, combining “celiac disease” with the terms “biomarker”, “i-FABP”, “citrulline”, “gluten immunogenic peptides”, “zonulin”, “calprotectin”, “microbiome” and “multi-omics”. Priority was given to pediatric studies and to publications from the last decade; reference lists of retrieved articles were screened manually. This work is a narrative rather than a systematic review, and no formal risk-of-bias assessment was performed. A comparative overview of current and emerging biomarkers, with each classified as current or emerging, is provided in Table 1.

**Table 1:** Current and emerging biomarkers in pediatric celiac disease.

Biomarker	Category	Sample	Clinical application	Advantages	Current limitations
Anti-TG2 IgA	Current	Serum	Initial diagnosis	High sensitivity and specificity	Reduced sensitivity in IgA deficiency; poor correlation with mucosal healing
EMA-IgA	Current	Serum	Diagnostic confirmation	Very high specificity	Less useful for monitoring
HLA-DQ2/DQ8	Current	Blood	Exclusion of genetic predisposition to CD	Excellent negative predictive value	Low positive predictive value
i-FABP	Emerging	Serum, urine	Early enterocyte injury; monitoring recovery	Reflects epithelial damage rapidly	Cut-offs not standardized; not CD-specific
Citrulline	Emerging	Serum	Enterocyte mass assessment	Correlates with villous atrophy	Confounded by renal dysfunction and other enteropathies
Gluten immunogenic peptides	Emerging	Stool, urine	GFD adherence monitoring	Objective detection of recent gluten intake	Short detection window
Zonulin (zonulin-related proteins)	Emerging	Serum, stool	Intestinal permeability	Non-invasive	Limited analytical and disease specificity; assay variability
Fecal calprotectin	Emerging	Stool	Intestinal inflammation	Easy non-invasive assessment	Low specificity for CD
HMGB1	Emerging	Serum, stool	Immune activation	Potential activity marker	Limited pediatric validation
Cytokines (IL-2, IL-8, IL-17A, IL-21)	Emerging	Serum	Immune activation; disease activity	Reflect immunopathogenesis; IL-2 rises within hours of gluten	No standardized assays; short half-lives
MicroRNAs	Emerging	Serum, plasma, urine	Early diagnosis; prognosis	High stability	Mostly experimental; methodological heterogeneity
Transcriptomic signatures	Emerging	Blood, tissue	Disease stratification	May identify molecular endotypes	High cost; limited availability
Gut microbiome-derived markers	Emerging	Stool	Risk assessment	Supports precision medicine	High interindividual and regional variability
Metabolomics/lipidomics	Emerging	Serum, stool	Disease phenotyping; preclinical prediction	Comprehensive molecular profiling	Research use only

“Current” biomarkers are those included in the ESPGHAN 2020 diagnostic guidelines (8); “emerging” biomarkers are investigational or adjunctive tools not yet recommended for routine diagnosis or follow-up. The sample column indicates the biological material in which each biomarker has been measured in published studies. Anti-TG2 IgA, IgA antibodies against transglutaminase 2; CD, celiac disease; EMA-IgA, endomysial IgA antibodies; GFD, gluten-free diet; HLA, human leukocyte antigen; HMGB1, high-mobility group box 1; i-FABP, intestinal fatty acid-binding protein (FABP2); IL, interleukin.

## 2 Emerging biomarkers in pediatric celiac disease

### 2.1 Blood-based biomarkers of enterocyte injury and immune activation

Intestinal fatty acid-binding protein (i-FABP), a small cytoplasmic protein expressed in mature villus-tip enterocytes, is rapidly released into the circulation following epithelial injury, making it a direct marker of ongoing intestinal damage rather than immune activation. Functionally, i-FABP (FABP2, approximately 15 kDa) binds long-chain fatty acids and takes part in their intracellular uptake and trafficking. Because of its low molecular weight and cytosolic location, it leaks from enterocytes as soon as their membranes are damaged and is cleared by the kidneys, which also allows it to be detected in urine (16). Serum i-FABP is consistently elevated in children with newly diagnosed CD, correlates with Marsh-graded villous atrophy severity, and declines rapidly after starting a GFD, often before conventional serology normalizes (16–18). Experimental gluten-challenge studies show i-FABP rising within days of gluten ingestion, before changes in anti-TG2 titers (19). A recent single-center pediatric study from Uzbekistan likewise reported significantly elevated i-FABP in children with newly diagnosed CD compared with healthy controls, together with increased fecal zonulin (20). However, i-FABP is not CD-specific, being also elevated in inflammatory bowel disease, intestinal ischemia, and necrotizing enterocolitis, and diagnostic thresholds vary across assays, limiting its use as a standalone test (21).

Plasma citrulline, synthesized almost exclusively by mature enterocytes, reflects functional enterocyte mass rather than immune activity. Citrulline is a non-protein amino acid formed from glutamine in enterocyte mitochondria and converted to arginine mainly in the kidney. Because it is not incorporated into proteins, its plasma concentration depends chiefly on intestinal production and renal clearance (21). Concentrations fall in proportion to villous atrophy severity and rise gradually over months of GFD as the mucosa regenerates, closely paralleling histological recovery (21). Citrulline may help distinguish active villous atrophy from potential CD with preserved histology, though renal dysfunction, bowel resection, and other enteropathies can confound results, and pediatric reference ranges remain unestablished.

Cytokines reflect the coordinated innate and adaptive immune response to gluten. Interleukin-2 (IL-2) and interferon-*γ* are secreted by gluten-specific CD4^+^ T cells after they recognize deamidated gluten–HLA-DQ complexes. Interleukin-15, produced by epithelial and lamina propria cells, licenses intraepithelial cytotoxic lymphocytes to kill enterocytes. IL-21 and IL-17A sustain the T-helper 1/T-helper 17 inflammatory response, and IL-8 recruits neutrophils (1, 22). IL-2 rises within hours of gluten ingestion and is currently the best-characterized acute-response cytokine in CD: multiplex studies have shown elevated serum IL-2 in the majority of treated patients after a single gluten challenge, with the magnitude of the rise correlating with symptom severity (22, 23). Cytokine release after bolus gluten challenge also discriminates CD from self-reported non-celiac gluten sensitivity, supporting a diagnostic role in patients already on a GFD (24). IL-8, IL-17A, IL-21, IL-6, and tumor necrosis factor-α (TNF-α) are similarly elevated during active disease and generally correlate with anti-TG2 titers and villous atrophy severity; persistent elevation despite normalized serology may indicate incomplete mucosal recovery (24). Clinical use remains limited by short half-lives, inter-individual variability, and the lack of standardized assays (24).

High-mobility group box 1 (HMGB1), a nuclear damage-associated molecular pattern released from injured epithelium, is upregulated at diagnosis in children with CD and appears to reflect the severity of histological damage (25). HMGB1 is a non-histone chromatin-binding protein that is passively released by necrotic cells or actively secreted by activated immune cells. Outside the cell it acts as an alarmin through Toll-like receptor 4 (TLR4) and the receptor for advanced glycation end products (RAGE), amplifying innate inflammation (25). It may therefore offer complementary information to serology in patients with persistent symptoms despite normal antibodies. HMGB1 is not CD-specific, and pediatric evidence remains limited to small single- and multicenter cohorts.

Collectively, these blood biomarkers capture complementary dimensions—epithelial injury, enterocyte mass, and immune activation—that conventional serology cannot. None yet meets criteria for a standalone diagnostic test, but combinations, particularly i-FABP and citrulline, show growing promise for pediatric use.

### 2.2 Stool- and urine-based biomarkers

Gluten immunogenic peptides (GIPs), digestion-resistant gluten fragments detectable in stool and urine, provide objective evidence of recent gluten exposure, identifying both intentional and inadvertent intake even when anti-TG2 antibodies are still normal. Fecal GIPs are more sensitive than dietary questionnaires for detecting poor GFD adherence, though their short detection window (2–4 days in stool, 24–48 h in urine) means a negative result does not exclude intermittent lapses or ongoing mucosal injury (11, 14). The relevance of this sensitivity is underlined by a placebo-controlled dose-escalation study in treated adults, in which urinary GIPs became detectable in almost half of the patients after a single 50-mg gluten dose, independently of symptoms (26). Current evidence supports GIPs primarily as a tool for objective adherence monitoring rather than assessment of mucosal healing (27).

Zonulin, a physiological regulator of intestinal tight junctions, reflects epithelial barrier integrity (28). Elevated serum and fecal zonulin have been reported in newly diagnosed pediatric CD, consistent with the concept that barrier dysfunction precedes overt villous atrophy (20). However, commercial enzyme-linked immunosorbent assays lack analytical specificity, recognizing properdin and other zonulin-related proteins rather than the pre-haptoglobin-2 precursor itself (28), and elevated levels occur in numerous inflammatory and metabolic conditions, limiting disease specificity. Zonulin should currently be regarded as a research biomarker rather than a validated diagnostic tool.

Fecal calprotectin, a marker of neutrophil-driven intestinal inflammation, is elevated in a proportion of untreated CD patients, particularly those with prominent gastrointestinal symptoms, and typically declines after GFD initiation (29). However, its correlation with histological severity and serological activity is weak, and elevated fecal calprotectin occurs across numerous inflammatory gastrointestinal disorders, limiting its specificity for CD.

Overall, stool- and urine-based biomarkers provide complementary information on gluten exposure and barrier or mucosal inflammation. GIPs are closest to routine clinical implementation, while zonulin and calprotectin remain adjunctive candidates requiring methodological standardization and pediatric validation.

### 2.3 Microbiome and multi-omics biomarkers

CD pathogenesis extends beyond gluten-specific immunity to involve host genetics, microbiota, and environmental exposures. Although HLA-DQ2/DQ8 is the strongest genetic determinant, only a minority of genetically susceptible children develop CD, implicating environmental modifiers of gluten tolerance. Pediatric studies show intestinal dysbiosis in active CD, including reduced diversity, depletion of *Bifidobacterium* and *Lactobacillus*, and expansion of pro-inflammatory taxa such as *Bacteroides* and Proteobacteria—changes that may promote increased intestinal permeability and mucosal inflammation (30). Some of these differences appear to be shaped by the host genotype. In infants with a first-degree relative with CD, carriers of the high-risk HLA-DQ2 genotype already had higher proportions of Firmicutes and Proteobacteria and lower proportions of Actinobacteria, including *Bifidobacterium*, than low-risk infants in early life (31).

The microbiota can also directly modify how immunogenic gluten is. *Pseudomonas aeruginosa* isolated from the duodenum of patients with CD used its elastase to generate gluten peptides that activated gluten-specific T cells and crossed the epithelial barrier more efficiently. By contrast, *Lactobacillus* strains from healthy controls degraded these peptides into less immunogenic fragments (32). Microbial metabolism also offers stool- and urine-based readouts that may be more practical than taxonomic profiling. In children with treated CD, fecal and urinary volatile organic compounds, short-chain fatty acids, and free amino acids differed from those of controls despite at least 2 years on a GFD, indicating that the diet alone does not fully normalize the microbiota–metabolome axis (33).

Prospective birth-cohort metagenomic analyses in genetically at-risk infants have identified shifts in microbial strains, pathways, and metabolites detectable up to 18 months before CD onset, supporting a potentially pathogenic rather than purely secondary role for the microbiota (34). Whether these signatures are transferable across populations remains untested; regional differences in diet and early-life exposures mean that microbiome-based biomarkers will require validation in non-European cohorts, including Central Asia.

High-throughput multi-omics approaches—including transcriptomics, proteomics, and lipidomics—characterize molecular networks rather than single markers. In children, RNA sequencing of duodenal biopsies has revealed gene-expression changes related to immune response, nutrient transport, and epithelial barrier function. These changes were present even in children whose first biopsy was non-diagnostic, suggesting that expression profiling could complement histopathology in equivocal cases, although it appears less suitable for prognosis (35). Gluten-specific CD4^+^ T cells, although rare in peripheral blood, represent a highly disease-specific signature that can be detected even during a GFD using HLA-DQ-gluten tetramers and multiplex cytokine profiling (36), although these techniques remain technically demanding. Circulating and urinary microRNAs are stable and can be measured using minimally invasive approaches; several have been associated with mucosal inflammation and disease phenotype, but methodological heterogeneity limits clinical translation (37). Nuclear magnetic resonance–based metabolomic profiling of serum and urine showed that most patients with potential CD (24 of 29) shared the metabolic profile of overt CD, suggesting that metabolic changes precede villous atrophy and could help stratify this group (38). Lipidomic studies have demonstrated altered phospholipid and lipid-metabolism profiles months before gluten introduction in at-risk infants, suggesting that metabolic dysregulation may precede overt immune activation (39).

None of these approaches is yet ready for routine clinical use, but they represent a promising direction for preclinical risk prediction and individualized management. Their most realistic near-term applications are risk stratification of genetically predisposed infants, identification of children with potential CD who are likely to progress to villous atrophy, and objective assessment of mucosal and metabolic recovery on a GFD. Reaching these goals will require standardized sampling and analytical pipelines, integration with clinical and serological data, and validation in ethnically and geographically diverse pediatric cohorts.

## 3 Discussion

The expanding landscape of emerging biomarkers reflects a shift in pediatric CD research from conventional diagnosis toward precision medicine. Rather than replacing established serology, these biomarkers provide complementary information on epithelial injury, immune activation, intestinal permeability, gluten exposure, and host-microbiome interactions.

Among currently available candidates, i-FABP is one of the most promising indicators of enterocyte injury and mucosal recovery, while gluten immunogenic peptides represent one of the most clinically applicable tools for objective monitoring of GFD adherence. Recent pediatric work has extended i-FABP (FABP2) measurement to urine, raising the prospect of fully non-invasive sampling in children: urinary i-FABP was significantly higher in children with active CD than in controls, although discriminatory performance was only moderate and levels in children already established on a GFD did not differ significantly from either group (18, 40). Urinary sampling therefore appears technically feasible but is not yet validated against histology.

Important challenges remain. Most emerging biomarkers lack standardized assays, universally accepted reference ranges, and prospective pediatric validation; their performance is influenced by age, disease stage, dietary status, genetic background, and coexisting inflammatory disorders, limiting cross-study comparison. These gaps are especially relevant in Central Asia, where pediatric biomarker data remain scarce despite growing recognition of CD and considerable genetic and environmental diversity (3, 4, 20).

In the context of routine pediatric practice in Central Asia, the combination of anti-TG2 IgA with a marker of enterocyte injury such as i-FABP may represent a potentially useful and relatively accessible strategy, provided that assay availability, analytical costs, and pediatric reference ranges are established locally. However, i-FABP should currently be considered complementary to, rather than a replacement for, established serological testing and the ESPGHAN diagnostic pathway.

No single biomarker is currently accurate and affordable enough to replace the ESPGHAN diagnostic algorithm (8). The greatest clinical benefit is likely to come from multimarker strategies combining serology with indicators of intestinal injury, immune activation, and recent gluten exposure, particularly for patients with equivocal serology, selective IgA deficiency, potential or seronegative CD, or those already on a GFD (10, 12). Validated biomarker panels could also provide objective outcome measures for therapeutic trials, reducing reliance on repeated biopsy (41).

Looking forward, future management is expected to integrate genetic susceptibility (HLA-DQ2/DQ8), epithelial injury (especially i-FABP), enterocyte mass (citrulline), permeability (zonulin), immune activation (cytokines, HMGB1), dietary adherence (GIPs), and molecular signatures from transcriptomics, lipidomics, and microbiome analysis. Advanced multi-omics biomarkers should currently be considered primarily for use in tertiary centers and research settings, where analytical infrastructure and standardized protocols are available. Pediatric-specific reference ranges and longitudinal validation, particularly in regions where awareness of CD remains limited, remain essential prerequisites, since biomarker kinetics may differ substantially between early and late diagnosis.

In summary, emerging biomarkers might reshape pediatric CD management by complementing conventional serology and providing direct information on intestinal injury, immune activation, and gluten exposure. i-FABP and GIPs show the greatest promise for improving early diagnosis, follow-up monitoring, and assessment of GFD adherence in children, but most emerging biomarkers require multicenter pediatric validation, assay standardization, and age-specific reference ranges before routine clinical implementation.
